# Analysis of Normal-Tumour Tissue Interaction in Tumours: Prediction of Prostate Cancer Features from the Molecular Profile of Adjacent Normal Cells

**DOI:** 10.1371/journal.pone.0016492

**Published:** 2011-03-30

**Authors:** Victor Trevino, Mahlet G. Tadesse, Marina Vannucci, Fatima Al-Shahrour, Philipp Antczak, Sarah Durant, Andreas Bikfalvi, Joaquin Dopazo, Moray J. Campbell, Francesco Falciani

**Affiliations:** 1 School of Biosciences and IBR, University of Birmingham, Edgbaston, United Kingdom; 2 Department of Epidemiology & Biostatistics, University of Pennsylvania School of Medicine, Philadelphia, Pennsylvania, United States of America; 3 Rice University, Houston, Texas, United States of America; 4 Bioinformatics Department, Centro de Investigación Príncipe Felipe, Valencia, Spain; 5 Institute of Biomedical Research, School of Medicine, The Birmingham University, Birmingham, United Kingdom; 6 Computer Science Department & Biomedical Engineering Program, Instituto Tecnológico y de Estudios Superiores de Monterrey, Nuevo Leon, Mexico; 7 Department of Pharmacology and Therapeutics, Roswell Park Cancer Institute, Buffalo, New York, United States of America; 8 INSERM U920, Talence, France; 9 University Bordeaux I, Talence, France; Fondazione Telethon, Italy

## Abstract

Statistical modelling, in combination with genome-wide expression profiling techniques, has demonstrated that the molecular state of the tumour is sufficient to infer its pathological state. These studies have been extremely important in diagnostics and have contributed to improving our understanding of tumour biology. However, their importance in in-depth understanding of cancer patho-physiology may be limited since they do not explicitly take into consideration the fundamental role of the tissue microenvironment in specifying tumour physiology. Because of the importance of normal cells in shaping the tissue microenvironment we formulate the hypothesis that molecular components of the profile of normal epithelial cells adjacent the tumour are predictive of tumour physiology. We addressed this hypothesis by developing statistical models that link gene expression profiles representing the molecular state of adjacent normal epithelial cells to tumour features in prostate cancer. Furthermore, network analysis showed that predictive genes are linked to the activity of important secreted factors, which have the potential to influence tumor biology, such as IL1, IGF1, PDGF BB, AGT, and TGFβ.

## Introduction

The application of functional genomics technologies, particularly gene expression profiling, has provided the scientific community with the tools to characterize the molecular state of cells and tissues at a genome level. These technologies coupled with the ability to dissect specific cell types from a complex tissue have created an unprecedented opportunity to characterise the molecular identity of specific cell types in the context of a complex tissue [1]. Following this approach, gene expression profiling have been applied to generate the transcriptional profile of tumour cells that are predictive of both tumour features and clinical outcome in a variety of human cancers [2]. Many genome-wide studies however are often analyzed not taking explicitly into consideration that components of the extra-cellular matrix (ECM) (matrix proteins, soluble grow factors and chemokines) secreted by normal cells, adjacent to the tumour site, heavily influence the biology of the tumour. Recently, stromal cells have emerged as primary candidates for playing a role into normal-tumour cell interaction [3]. These cells secrete most of the enzymes involved in ECM breakdown, for example they produce growth factors that have a role in controlling tumour cell proliferation, apoptosis, and migration. They also secrete pro-inflammatory cytokines involved in chemoattraction and activation of specific leucocytes and therefore play a role in determining inflammatory responses [4]. Growth factors and cytokines are also involved in the neoplastic transformation of cells, angiogenesis, tumour clonal expansion and growth, passage through the ECM, intravasation into blood or lymphatic vessels and the non-random homing of tumor metastasis to specific sites. Many of these factors are also secreted by normal epithelial cells, immune cells and endothelial cells in proximity of the tumour mass. It has also been shown that the stroma may impact on the response to anti-tumour therapy. Indeed, the presence of CD11b+ leucocytes confers resistance to anti-angiogenesis therapy [5].

Furthermore, pre-treatment of the stroma with anti-angiogenesis molecules prior to tumour implantation in mouse tumour models may paradoxically increase tumour development [6], [7]. This illustrates that the quality of the tumour stroma may significantly influence tumour development.

The importance of the micro-environment in determining the onset and progression of cancer arises the question whether it may be possible to predict the patho-physiology and clinical outcome of the tumour from specific components of the molecular state of normal cells. If possible, we would expect these molecular signatures to represent important components of cell-cell cross-talk involved in specifying the development of cancer.

We addressed this question by developing statistical models based on a genome wide profiling of normal tissue adjacent the tumour and identifying aspects that are predictive of cancer features.

We have analyzed two different prostate cancer microarray datasets available in the public domain [8], [9]. We show that in both datasets the molecular state of cells adjacent to the tumour is predictive of clinically relevant cancer features. These pathways are informative molecular signatures and represent pathways involved in the production and response to secreted factors.

These findings support the potential relevance of normal tissue biopsies in the diagnosis and prognosis of prostate cancer. This approach also provides a generally applicable analysis strategy to identify key pathways involved in cell to cell communication.

## Results

### Statistical modeling establishes a link between the molecular state of normal cells and tumor histo-pathological features

The initial objective of our analysis was to test whether the molecular profile of normal cells is predictive of cancer features. We initially considered two important aspects of prostate tumour physiology: the degree of organization of tumour cells defined by a histo-pathological scoring system called Gleason score, and the ability of tumour cells to penetrate the organ capsule summarized by a binary histo-pathological score called capsular penetration. The level of differentiation of tumour cells measures the tendency of cells to aggregate in glandular-like structures that are reminiscent of the organization of the normal tissue. The Gleason score can be used to define two main classes. The first is characterized by low-grade tumours that display a highly organised structure (correspondent to a score below or equal to 6) whereas a second class is characterized by high-grade tumours cells that are dispersed in the matrix and do not show a tendency to form glandular-like structures (correspondent to a score above or equal to 7). By contrast capsular penetration describe the extent to which cells have evaded the capsule that surrounds the prostate.

Our analysis aimed to link the molecular profile of normal cells to differentiation level (low versus high differentiation) and capsular penetration (positive versus negative). This was achieved through the development of statistical models that were based on the molecular profile of normal cells and predictive of the sample classes, specifically Gleason score and capsular penetration.

**Figure 1:**
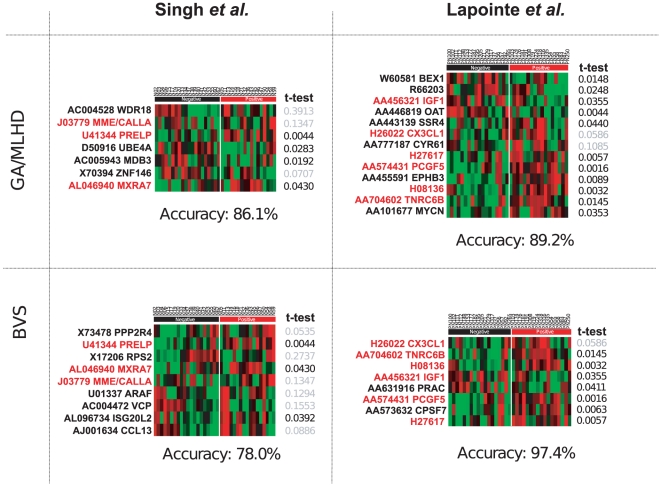
Multivariate Models for Capsular Penetration using Normal data. The figure shows the heat maps representing the expression profile of genes selected by the GA and BVS models in both Lapointe and Singh datasets from the normal tissue data. Each quadrant in the figure represents a combination of a modelling approach and a specific dataset. Genes present in GA-MLHD and BVS for the same dataset are highlighted in red. Accuracy is reported below each heatmap. GeneBank accession number and gene symbol are shown on the left side of the heatmap. Brighter green or red colours in heatmaps represent lower or higher relative expression respectively. t-test p-value is shown for comparison with the differential expression criteria commonly used in univariate variable selection approaches.

For this purpose we applied two different multivariate modelling approaches (GA-MLHD and BVS methods) to two independent datasets developed by Singh et al. [9] and by Lapointe et al. [8]. The two statistical modelling approaches are designed to search for multi-gene markers that maximise the distinction between sample classes. Using these methods, we have developed representative models that were predictive of tumour features by means of the gene expression profile of normal cells. Classification accuracy and size of these models were comparable to the ones developed using the molecular state of tumour cells (**Table 1**). Representative models developed with the BVS and GA-MLHD methods represent optimal predictive subsets that are based on a very similar number of genes and have a high degree of overlap at the gene level, suggesting that our results are independent of the methodology used (**Figure 1** **and Figures S1, S2, and S3**). Consistent with the relatively small degree of overlap between the microarray platforms (< = 8%, see the Data Processing section in the Text S1 for details), the representative models developed from the two independent datasets have no genes in common.

**Table 1:** Accuracy, size, and gene content of representative models developed from normal and tumour data.

*Class+Tissue* *Dataset*	*GA-MLHD*	*Acc*	*BVS*	*Acc*
*CP+N*Singh *et al.*	WDR18, ZNF146, MBD3, UBE4A, PRELP*, MXRA7*, MME/CALLA*	86.1 (7)	RPS2, CCL13, VCP, PRELP*, PPP2R4, ISG20L2, MXRA7*, ARAF, MME/CALLA*	78.0(9)
*CP+T*Singh *et al*.	LUZP1, SORL1, TMSL8*, HYOU1, ST14, TALDO1*, DGCR6L	97.4 (7)	TMSL8*, RPL35, HIST1H2BK, KRT8, RAB1A, TSPAN1, TALDO1*, PDLIM5, GADD45G, GDF15	92.0(10)
*GS+N*Singh *et al*.	BZRPL1, TEGT*, IDH3B, MID1, D83779, PTGDS*, PFDN5, PTGDS*	89.7 (8)	HBA1/2, PABPC1/3, TEGT*, PRSS22, DNAJC4, PTGDS*, PNPLA2, USP9X, PTGDS*	92.5(9)
*GS+T*Singh *et al*.	TMSB4X/L3, SLC6A7, AA524802, ABCC10, INHBB, SULT2B1, PHYHIP, SLC1A5, ACPP*, C7, ACPP*, NR4A1*	91.6 (12)	VIM, R42599, ARF1, RBM3, EIF4G2, ACPP*, VEGF, SPARCL1, COL4A2, HLA-DPB1, DSTN, UBB, ACPP*, NR4A1*	90.0(14)
*CP+N*Lapointe *et al*.	H27617*, PCGF5*, IGF1*, OAT, EPHB3, BEX1, C12orf56, H08136*, IDH3G, CYR61, TNRC6B*, CX3CL1*, MYCN	89.2 (13)	H27617*, FLJ12529, PCGF5*, PRAC, IGF1*, H08136*, TNRC6B*, CX3CL1*	97.4(8)
*CP+T*Lapointe *et al*.	MT1X*, R20199*, NEBL, ACSL3, CXCL14*, AI018472	96.5 (6)	AA420602, H19, MT1X*, CIP29, R20199*, PLGLB2, ZNF533, CXCL14*, NAT1	100.0(9)
*GS+N*Lapointe *et al*.	FOLR1, APOD, NALP2*, CLSPN, N39101, ISL1*, KITLG, N46872, APOD, KBTBD10*, ZNF185*, AA699363, FUT8, KLK2*	93.8 (14)	NALP2*, ISL1*, KIAA1244, KBTBD10*, ZNF185*, AI018026, KLK2*, RERG	97.1(8)
*GS+T*Lapointe *et al*.	MOCOS, ITGBL1*, PLEKHH2, WDR72*, DUSP8*, RBM12B, MCOLN3	94.4 (7)	ITGBL1*, S100A1, KBTBD10, AA699944, WDR72*, MTMR9, DUSP8*, PUNC	97.1(8)

Accuracy (Acc) are expressed in percentage and model size are shown in brackets. Marked genes in bold and asterisk appear in both methods (GA-MLHD and BVS). Dataset is indicated. CP+N – Capsular Penetration class from Normal data, CP+T – Capsular Penetration Tumour, GS+N – Gleason Score Normal, GS+T – Gleason Score Tumour.

**Figure 2:**
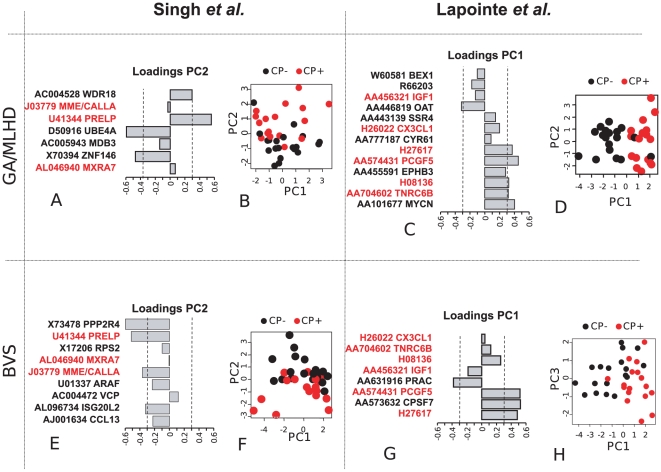
Principal component representation for Capsular Penetration using Normal Data. The figure shows the result of a PCA representing sample separation on the basis of the expression in normal tissue of genes selected by the modelling procedures. Each quadrant in the figure represents a combination of a modelling approach and a specific dataset. Each quadrant contains a 2D plot representing the separation of capsular penetration negative (black close circles) and positive (red close circles) samples (plots B, D, F and H) and a bar chart (plots A, C, E and G) representing the PC loadings (x axis) for each gene component (y axis). Note that PC loadings represent the contribution of every gene to class separation. Dashed lines delimitated genes with larger contribution that are discussed in the manuscript. Genes present in GA-MLHD and BVS for the same dataset are highlighted in red.

Further analysis of the relative contribution of the individual genes to the sample separation was performed using a principal component analysis (**Figure 2**). This approach has revealed that genes involved in cell communication pathways are predictive of capsular penetration. Within the gene set selected by the GA in the normal tissue dataset, a combination of higher expression of the gene *PRELP* and a lower expression of the genes *UBE4A*, *ZNF146* in the normal cells was predictive of tumour capsular penetration. In the gene set developed by applying the BVS procedure on the normal tissue dataset, a high expression of *PPP2R4*, *PRELP*, *CALLA*, *ISG20L2* was predictive of tumour capsular penetration. The models developed from the Lapointe dataset revealed that lower expression of *OAT* and higher expression of *PCGF5* and *MYCN* in the GA model and lower expression of *IGF1* and *PRAC* and higher expression of *PCGF5* and *CPSF7* are predictive of capsular penetration.

The link between normal and tumour shown in this analysis is also supported by a univariate analysis which we have performed using a broad spectrum of available methodologies (**Figure S4**).

### Specificity of gene signatures predictive of cancer histo-pathological features

Adjacent normal and tumour tissues are morphologically distinct. However, they show a degree of molecularly similarity which is in part a consequence of sharing the same micro-environment [10]. We therefore wondered whether the predictive models we have developed from normal epithelial cells represent a molecular signature that is specific to normal tissue or whether the expression of the predictive genes in tumour cells may also be predictive of tumour features. In order to address this hypothesis, we took genes selected by our modelling strategies developed from the normal tissue datasets and tested whether their expression in the tumour issue was predictive of cancer features.

**Figure 3:**
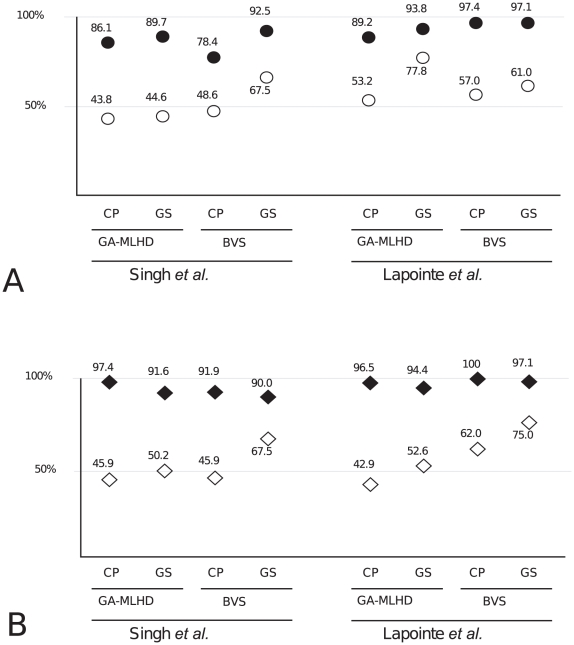
Accuracy and Tissue specificity of representative models. The predictive accuracy of the models developed using normal tissue (panel A, filled circles) is comparable to those models developed using tumour tissue (panel B, filled diamonds). When models developed using normal tissue are trained and tested using data from tumour tissue, the prediction power is decreased considerably (empty circles). Likewise, tumour models trained and tested with data from normal tissue are also non predictive (empty diamonds).

We also challenged the prediction accuracy of models developed from the tumour data by performing the corresponding comparison in the normal dataset. In both cases, the prediction accuracy of the models is close to 50% (which correspond to the expected accuracy of a random guess) (**Figure 3**). This analysis therefore shows that the molecular signatures we have identified are specific for the tissues (normal or tumour) they have been selected to represent.

### Functional networks linked to predictive signatures representing normal epithelial cells expression profiles include important cytokine and growth factor signals

In order to facilitate the biological interpretation of the genes represented in our statistical models we used the IPA analysis software to perform an in depth analysis at the network level. To ensure our analysis covered the full spectrum of possible solutions, we used as input to the IPA software the list of genes represented in the collection of predictive models identified from the normal tissue by the GA procedure. These covers a wider spectrum of the solution space respect to the representative models described above (**Figure 1** and **2**) and represent 239 and 259 genes for Singh and Lapointe datasets respectively. In this analysis we focused on Capsular penetration because of its clinical and prognostic relevance. The network analysis was performed independently in the two datasets and the most significant networks (statistically significant and with >50% target genes represented in the network) were selected for further analysis.

In both datasets, predictive genes were part of networks linking extracellular molecules such as the pro-inflammatory cytokine *IL1β*, the pro-metastatic chemokines *CX3CL1* and *CCL20* and the growth factors *IGF1*, *TGFβ* and *PDGF BB* with the activity of the nuclear transcription factors *NFKb*, *HF4A*, *TP53*, and *MYC*.

**Figure 4:**
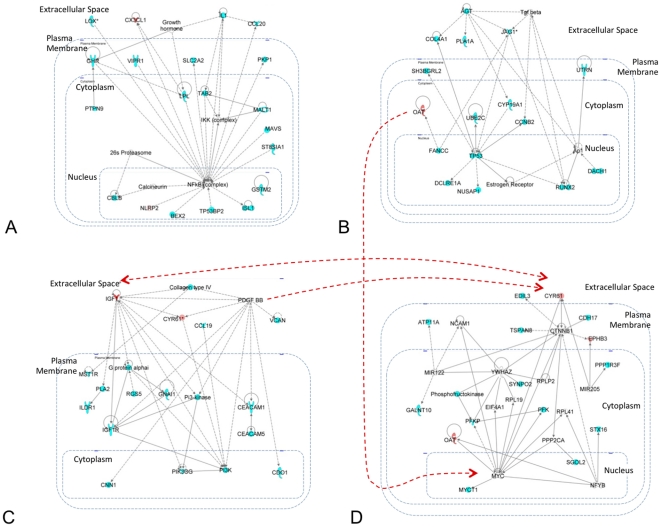
Functional networks representing known interaction between genes expressed in normal tissue and selected in the models predictive of capsular penetration. The figure represents the four most significant networks selected by the IPA software. Genes represented by blue shapes are present in the collection of models collected by the GA-MLHD procedure. Genes represented with red shapes represent genes in the collection of models but also included in the representative most predictive models. Genes in the networks are arranged by cellular localization (extracellular, membrane, cytoplasm and nucleus). Note that the IPA software search for statistically significant sub-networks of a given maximum size to simplify their visualization. Nevertheless, in this case these are linked as indicated by red dashed arrows connecting specific network components.

**Figure 4** describes the most significant networks identified by the IPA application representative of the models based on the molecular state of normal cells and predictive of capsular penetration in the Lapointe et al. dataset (see **Table S2** for the full list of significant networks identified by IPA). **Figure 4A** shows a network represented by the interaction between the pro-inflammatory cytokine *IL1β* and the transcription factor *NFkB*. **Figure 4B–D** represent three interconnected sub-networks which involve the interaction between several growth factors genes and the transcription factors P53 (*TP53*) and C-MYC (*MYC*). More specifically, **Figure 4C** represent a network including the growth factors *IGF1*, its receptor *IGF1R* and *PDGF BB*. **Figure 4B** represents the interaction between the extracellular factors Angiotensin (*AGT*), the growth factor *TGFβ* and the Notch receptor ligand Jagged (*JAG*). **Figure 4D** on the other hand represents genes that are either directly or indirectly connected to the transcription factor c-myc (*MYC*).

The top four most significant networks identified from the Singh dataset (**Figure S5**) represent genes connected to the same cytokines and growth factors identified in the Lapointe dataset. This interesting observation suggests that, despite the limited amount of overlap at the gene level, models derived from the two dataset may represent functionally similar molecular networks.

### Expression of predictive cytokines, growth factors and their receptors in Prostate Cancer progression

In order to improve understanding of the biological significance of the IPA networks we analysed the expression of genes in different stages of prostate cancer progression. We focused the investigation on a small subset of 20 genes representing the secreted factors included in the IPA networks and their receptors (**Table S3**).

With the purpose of limiting the interference of stromal cell contaminants, we selected a dataset representing a microarray analysis of seven types of normal and tumour epithelial cells populations, purified by laser-capture micro-dissection (LCM) reported by Tomlins et al. [11]. These included, normal prostate cells purified from healthy prostates (Nor), normal cells from benign prostate hyperplasia (BPH), normal cells adjacent the tumour (adj), tumour cells from prostatic intraepithelial neoplasia (PIN), tumour cells from low grade prostate carcinoma (L-PCA), tumour cells from high grade prostate carcinoma (H-PCA) and tumour cells from prostate cancer metastases (Meta).

**Figure 5:**
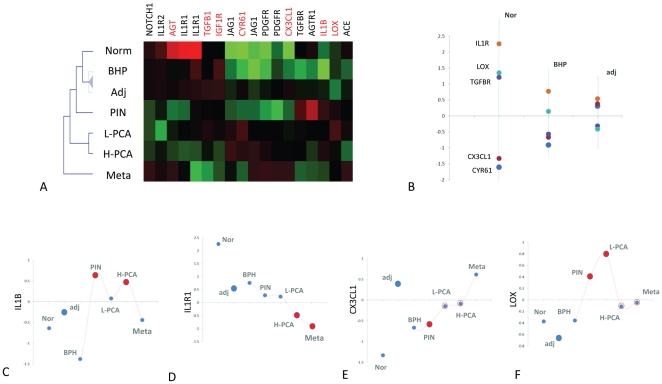
Analysis of LCM cell populations representative of prostate cancer progression. The figure represents the results of the analysis performed on the dataset developed by Tomlins et al. [11]. Different cell populations are labelled as follows. Normal cells (norm), normal cells adjacent the tumour (adj), benign prostate hyperplasia (MPH), low grade prostate carcinoma (L-PCA), high-grade prostate carcinoma (H-PCA) and metastatic cells (meta). **Panel A** shows a two-dimensional cluster analysis performed on the genes differentially expressed (*p<0.01*) across the seven LCM purified normal and tumour epithelial cell populations. **Panel B** represents the expression level (y axis) of genes differentially expressed between norm, adjacent and BPH (represented on the y axis). Levels of individual genes across all stages are presented in panels C-F and in **Figure S8**.

We hypothesize that since the 20 genes we selected were included in models highly predictive of tumour capsular penetration, they may also be differentially expressed during prostate cancer progression. We tested this hypothesis by comparing the seven LCM cell populations. We discovered that a surprising large proportion of these genes were differentially expressed (75% at p*<0.001* and 95% at *p<0.05*) (**Table S3**, **Figure 5**, **S7** **and** **S8**). Further support to the relevance of the gene expression signature we had identified came from the observation that the two dimensional cluster analyses performed using the matrix of differential gene expression profiles (average expression for each group), recapitulated the expected relationship between the different stages in the development of prostate cancer (**Figure 5A**). More precisely, normal cell populations clustered together followed by PIN and a cluster of L- PCA and H-PCA. The Metastatic cell group clustered aside.

Of relevance for understanding the biological basis of the predictive power of normal cells signature is the observation that normal cells adjacent the tumour showed significant differences in respect to Normal cells and BPH (**Figure 5B**). Five genes (*IL1R, LOX* and *TGFBR, CX3CL1* and *CYR61*) were differentially expressed between the three populations of normal cells. More specifically, normal cells adjacent to the tumour (Norm) were characterized by a lower expression of the tumour suppressor gene *LOX*, the receptors for interleukin 1 (*IL1R*) and *TGFβ* (*TGFBR*) and by a higher expression of the pro-tumour genes *CYR61* and *CX3CL1*.

We then examined the expression of individual genes across the different stages of tumour progression in relation to the networks identified by the IPA software (**Figure 4**).

The cytokine *IL1β*, identified by the IPA analysis as linked to the activation of the pro-metastatic chemokines *CX3CL1* and *CCL20* (**Figure 4A**), was up-regulated in the tumour cell populations PIN and H-PCA (**Figure 5A, and 5C**), whereas the expression of *IL1R1*, which mediated the activity of *IL1β*, follows an opposite trend (**Figure 5A, B and C**). The pro-metastatic chemokine *CX3CL1* was expressed at higher levels in adjacent cell population respect to PIN, L-PCA and H-PCA but not in Meta cells (**Figure 5E**). The expression of the *LOX* gene was found higher in all tumour cell populations relative to adjacent and normal cells (**Figure 5F**) consistent with the fact that higher expression of *LOX* has been associated to hypoxia-induced metastasis in breast, head, neck cancers [12], [13].

The expression of *AGT*, *TGFB* and *JAG1* were linked in a different IPA network (**Figure 4B**). The expression of Angiotensinogen (*AGT*) is higher in adjacent cells compared to PIN, L-PCA and H-PCA whereas *JAG1* follows an opposite trend (down regulated in adjacent cells respect to L-PCA, H-PCA and Meta). If angiotensinogen is produced at higher levels in adjacent cells one of the activating enzymes which convert the product of the *AGT* gene in angiotensin II (*ACE*) is instead higher in PIN and L-PCA, suggesting the potential for utilization in tumour cells at lower stages of prostate cancer development. The finding that *AGT* and *JAG1* have opposite trends supports the hypothesis that *AGT* may repress the expression of *JAG1* (**Figure S8 panels E and F**). This connection was reported by the IPA software (**Figure 4B**) but was supported by an endothelial cell culture experimental model [14]. These results are consistent with the hypothesis that this mechanism may also be relevant in prostate cancer.

A third IPA network represents the interaction between the tumour-promoting factors *IGF1, PDGF BB* and *CYR61* (**Figure 4C**). Although the expression of *PDGF* is constant in all cell populations, its receptor (*PDGFR*) is higher in H-PCA and Meta cell populations compared to adjacent cells. The expression of *CYR61* is higher in adjacent cells respect to PIN and Meta cell populations (**Figure S8**).

## Discussion

We have demonstrated that normal epithelial cell signatures are predictive of important features of prostate cancer. This finding has potential clinical implications as it may suggests that the molecular state of normal cells has prognostic value. At the molecular level, network analysis has revealed that our approach has the potential to identify genes involved in the disease pathogenesis. These include key genes encoding cytokines and growth factors expressed by normal epithelial cells and known to influence the biology of the tumour.

### Cytokine induced production of pro-metastatic chemokines

The network shown in **Figure 4A** and **Figure S5** represents signalling of the pro-inflammatory cytokine interleukin 1 (*IL1β*) through the activation of the *NFkB* complex. The IPA software linked *IL1β* to the expression of the known pro-metastatic chemokines *CX3CL1* [15] and *CCL20* [16] in an endothelial cell culture model [14].

Although induction of these chemokines by *IL1β* has not been demonstrated to date, several pieces of evidence support the relevance of this mechanism in prostate cancer progression. Voronov et al. [17] have shown that IL1β is required for tumour invasiveness and angiogenesis in a mouse breast cancer model and provided evidence for the same mechanism in prostate cancer. More recently, an Interleukin-1 receptor antagonist haplotype have been found to be associated with prostate cancer risk [18] suggesting that the results of the animal model may be relevant in a clinical setting.

The analysis of the LCM dataset showed that *IL1β* is expressed at higher levels in PIN and H-PCA than in adjacent cell populations whereas the latter expressed higher levels of the receptor (*ILR1*) (**Figure 5**).

Furthermore, normal epithelial cells express higher levels of *CX3CL1* respect to their tumour counterpart while its receptor (*CX3CR1*) is expressed in tumour cells [19]. This chemokine promotes migration of cancer cells and metastases formation in a number of cancers [20] including prostate [21]. The production of this chemokine by epithelial cells adjacent the tumour has therefore the potential to induce tumour cell migration. Similarly, prostate normal epithelial cells produce the chemokine *CCL20* and the expression of its receptor (*CCR6*) in prostate cancer cells has been recently found to be a predictor of tumour aggressiveness [22].

The network also includes *LOX*, which is represented as an indirect repressor of the NFKb complex [23] [24]. The biological role of LOX in cancer is complex. LOX has been reported to have tumour suppressor activity [25] and can inhibit proliferation of the prostate cancer cell line DU 145 via a mechanism involving interference with FGF2 binding and signalling cascade [23]. However, LOX has also been reported to have an important tumour promoting activity by favouring metastasis in breast, head, and neck cancers [12] [13]. The analysis of the LCM dataset has shown that the expression of the *LOX* is higher in all tumour cell populations (PIN, L-PCA, H-PCA and Meta) respect to adjacent cells. This may be consistent with the tumour-promoting role of LOX but it raises the question whether the amount of *LOX* produced by epithelial cells would be able to significantly affect tumour cells.

The hypothesis that IL1*β* may trigger the activation of pro-metastatic signals in normal epithelial cells is an interesting one. We have initially tested this hypothesis by treating the normal prostate cell line RWPE1 with recombinant IL1*β* and discovered that both CCL20 and CX3CL1 are significantly up regulated 6 and 24 hours after stimulation. LOX is instead only transiently up-regulated six hours after IL1*β* stimulation (**Figure S6**). This observation suggests that our hypothesis may be correct.

### Role of IGF1 and PDGF BB in Prostate Cancer development

The IPA software identified a network representing interactions with the growth factors *PDGF BB* and *IGF1* (**Figure 4C and** **S5B-C**).

The role of IGF1R in malignant transformation is well documented [26]. IGF1R is over-expressed by many tumour cell lines and targeted disruption of the IGF1R gene can abolish cell transformation.

PDGF BB has a dual role on prostate cancer development. It directly promotes tumour cell proliferation and invasion [27]. Platelet-derived growth factor induces proliferation of hyperplastic human prostatic stromal cells [27].

In addition, PDGF BB has been described as a potent inductor of angiogenesis and promotes pericyte recruitment [28]. Its activity is synergistic to IGF1 in promoting migration of human arterial smooth muscle cells [29].

The IPA network shows that *PDGF BB* and *IGF1* can transcriptionally activate *CYR61* [30] [31]. CYR61 is an extracellular matrix-associated protein that promotes adhesion, migration, proliferation, and angiogenesis. CYR61 is required for breast tumorigenesis and cancer progression [32] [33] and promote prostatic cell adhesion and proliferation [34] [35]. CYR61 also promotes invasion when tumor stroma is irradiated before tumor implantation in a model of skin cancer [36]. Relative to LCM normal (norm) cells, *CYR61* is up regulated in LCM BPH, normal cells adjacent to the tumor and both low and high-grade prostate carcinoma (**Figure 5B and S** **8**). The receptor of *PDGF BB, PDGFBR,* has a similar trend, which is consistent with its potential activator role.

Although protein measurements may be necessary to support this analysis, it is not unreasonable to hypothesize that adjacent normal epithelial cells may produce sufficient CYR61 to influence tumor cells.

### The expression of targets of TGFβ in the normal tissue predict tumour capsular penetration

The networks represented in **Figure S5D** and **4B** represent the connection between genes including models predictive of tumour capsular penetrations and TGF.

TGFβ has a complex role in tumour development. It can either promote or inhibit tumour development in a context dependent manner [37]. In normal epithelia and early stages of tumour development, TGFβ has role in regulating tissue homeostasis and is considered an anti-tumour factor preventing incipient tumours from progressing towards malignancy [5]. Furthermore, TGFβinhibits recruitment of pericytes to the vasculature, thus decreasing vessel maturation and flow which may also negatively impact on tumour development [38].

At later stages of tumour development, TGFβ has been shown to promote tumour development and metastases formation. Of particular relevance, TGFβ1 reverses inhibition of COX-2 with NS398 and increases invasion in prostate cancer cells [39]. The development of a pro-tumour activity of TGFβ in tumour progression is often associated to mutations, which eliminate the tumour suppressor activities of TGFβ and promote growth and invasion. Another pro-tumour effect of TGFβ is linked to induce immune system tumour tolerance [37]. Consistent with these findings, recent reports suggest that in prostate cancer TGFβ may be relevant therapeutic target [40] [39].

We found that in the LCM cell populations *TGFβ* is expressed at high levels in normal cells adjacent the tumour (**Figure 5** and **S7**). The predictive power of *TGFβ* response signatures in normal epithelial cells may therefore be the reflection of the amount of active TGFB present in the microenvironment that, at least in part may be produced by normal epithelial cells adjacent to the tumour.

### Angiotensinogen and Notch in Prostate Cancer

The network shown in **Figure 4B** involve the interaction between the Angiotensin precursor Angiotensinogen (*AGT*) and the Notch ligand *JAG1*. A functional Renin-Angiotensin system has been demonstrated in prostate cancer [41] [42]. In addition its canonical role in regulating blood pressure it is now recognized that Angiotensin can influence several growth factor pathways [41], including oncogene activation [43]. It has been recently shown to be a clinically relevant factor in the progression of prostate cancer and a potential avenue for treatment [41] [44] [45]. *AGT* is up regulated in normal epithelial cells adjacent the tumour compared to PIN and PCA (**Figure S8E**). This observation is consistent with a biological role of AGT production by normal epithelial cells.

The IPA network (**Figure 4B**) links *AGT* to the transcription of *JAG*, another factor known to have context-dependent effects on tumour development. In vascular cells, inhibition of Jagged promotes angiogenesis [46] favouring tumour growth. On the other hand Jagged 1 favours proliferation and expansion of prostate tumours [47].

In LMC cell populations tumour cells express higher levels of *JAG1* than adjacent normal epithelial cells (**Figure 5** **and **S7****). Hence, the effective contribution of normal cell expressed JAG1 on tumour development is unclear.

### Conclusions

Ultimately, our approach provides a way to identify molecular networks whose activity in normal epithelial cells is predictive of tumour features. Prostate cancer progression rates among so-called “favourable prognosis” localized tumours (e.g. Gleason score <6) are not precisely predicted by grade and stage at diagnosis. This lack of diagnostic accuracy has contributed to the conundrum of CaP over-screening and possibly over-treatment [48], [49]. A similar lack of prognostic accuracy is apparent when tumours recur during androgen depravation therapy. Greater diagnostic accuracy is thus imperative to distinguish at early stages indolent disease from aggressive phenotypes that can progress rapidly, and at late stage disease the lethal phenotypes.

Lessons from breast cancer studies have taught us that significant strides in diagnosis (and thus treatment) can be made by applying multiple genetic parameters to define disease with greater clinical resolution (reviewed in [50]). Such approaches have progressed less quickly in prostate cancer [51]. The current study suggests this need and gap in understanding can be met by utilizing gene expression signatures in the normal prostate tissue adjacent to the tumour as novel functional molecular biomarkers. In early stage disease especially identification and sampling of the tumour within the prostate gland can be highly challenging. Therefore it is highly advantageous and attractive to utilize gene expression signatures in the readily sampled normal tissue to make robust prognostic inferences concerning the tumour.

An important question is whether the statistical relationships we have discovered with our analysis reflect a key aspect of tumour microenvironment in which normal epithelial cells influence tumour biology. Although it is hard to provide a conclusive answer, the information available in the literature and our experimental validation in a normal epithelia prostate cell line (**Figure S6**) indicates that this may be a plausible hypothesis.

Despite the limited overlap at the gene level, mainly caused by our stringent pre-processing criteria (see methods section for details), the analyses we have performed on the two independent datasets provided similar results at the network level. This finding reinforces the validity of the overall analysis strategy. From a methodological standpoint our approach therefore has potential for formulating hypothesis on genes playing a role in controlling the development of cancer. The approach is general and likely to be applicable to other datasets for which tumour and adjacent normal samples are available.

## Materials and Methods

### Datasets

Our analysis is based on two independent large prostate cancer studies performed using different array technologies. In both studies, cells from tumour and adjacent normal tissues have been isolated and the extracted RNA has been hybridized on human microarrays for expression profiling. The first dataset used in our analysis is derived from a study performed by Singh *et al.* [9] where 52 samples of prostate tumours and adjacent normal tissues were collected from patients undergoing radial prostatectomy; then profiled using Affymetrix Genechip technology. The second dataset used was collected by Lapointe *et al.* [52] using cDNA arrays. In this study 41 paired normal and tumor specimens were removed from radical prostatectomy. Information about the histo-pathology of the tumor specimens (Gleason score and Tumor stage) was available for both datasets. Details of the data processing for both datasets are available in the supplementary material. After processing, the two datasets show relatively limited overlap at the gene level (up to 8%, **Table S1**). Consequently, we have opted for the two datasets to be analyzed separately.

### Statistical Modeling

#### Classification methods with univariate variable selection

Our analysis aims to identify molecular signatures predictive of two binary variables representing relevant features of tumor biology. These are the degree of differentiation of the tumor and the ability of the tumor to penetrate the organ capsule. To develop such signatures we have initially tested a univariate variable selection strategy based on an F test in combination with several classification methods (SVM, DLDA, PAMR, KNN, SOM) as implemented in the software application Prophet available in the Web based microarray analysis suite GEPAS [53]. This application uses a step-wise variable inclusion strategy to construct increasingly large models from a list of genes ranked by the value of the F statistics and implement a cross-validation strategy for error estimation. Results of this analysis are shown in **Figure S4**.

#### Classification methods with multivariate variable selection

In order to consider the effect of combinations of genes in the prediction of the histo-pathological variables we have used a statistical modeling approach in combination with multivariate variable selection procedures. In order to demonstrate that our results are independent of a particular methodology we developed and compared multivariate classification models obtained using two independent procedures. These methods differ for both the variable selection strategy and for the classification algorithms used. The first approach is a modification of the Genetic Algorithm –maximum likelihood discriminant analysis (GA-MLHD) method originally developed by Ooi and Tan [54]. This method uses a genetic algorithm approach for variable selection coupled to a MLHD functions classifier. The GA-MLHD methodology uses an initial random population of models (called chromosomes) and evolves from them highly accurate classifiers using a process that mimics natural selection. Accuracy was estimated as the proportion of guesses in test samples in a cross-validated manner. In our implementation [55] we have improved the error estimation strategy by using two-levels of cross-validations. The first level is used in the evolutionary step of the GA to evaluate the error in a subset of the dataset using a k-fold-cross-validation procedure (k = 5). The second level is used at the end of the evolutionary process, when all chromosomes are selected, to estimate the classification error as an average of the test error in 40 random splits (2/3 for training and 1/3 for testing) using the entire dataset. Model sizes of 5 were used, which showed a higher accuracy than 10 and 20 in average for 10,000 models. In addition, we have compared the results with models obtained using a Bayesian variable selection (BVS) approach that we have developed [56]. This method uses a multinomial probit model as classifier and Markov Chain Monte Carlo (MCMC) methods to search multivariate space for informative subsets of the variables. Error estimation and parameters settings have been described in [56]. Two runs were made for model sizes 10 and 20. The model with higher average accuracy was then chosen.

#### Selecting representative models

Both GA-MLHD and BVS modeling approaches provide a number of alternative models with comparable predictive value. These models tend to have a degree of overlap in their gene composition. It is therefore meaningful to select a single summary model that represents the most frequent solutions. In order to do so, for the GA-MLHD approach, we have used a forward selection procedure applied to the top 1% most predictive models selected using the GA procedure. In the case of BVS, we have tested models developed with the genes that were included in the subsets of variables most frequently visited by the MCMC search. The final list of models was generated by the union of the two chains with minimum average miss-classification error [56]. Interestingly, we have discovered that representative models developed with the GA-MLHD procedure largely overlaps with the pooled models from the BVS approach. We tested the overlap between models selected by the GA-MLHD procedure in the two datasets at different processing thresholds. The overlap between the top 50 raking genes (by frequency of inclusion in the model populations) in the model populations was always significant (see **Tables S4 and S5**).

### Tissue specificity of representative models

An important component of our strategy is to demonstrate that molecular signatures are tissue specific hence they are not representing a mere reflection of the overall similarity between normal and tumour tissues. The strategy to demonstrate the specificity of the gene signatures obtained with the multivariate variable selection strategy implemented in the GA-MLHD procedure is described below in two steps.

#### Step 1: development of representative models

Expression data from the normal tissue samples are split between training and test sets (respectively 2/3 and 1/3 of the original dataset). The training set is used to develop a classification model to predict cancer features with a cross-validation strategy. Once the representative models have been developed their classification accuracy is estimated on the test set.

#### Step 2: Specificity test

Expression data from the tumour tissues samples are split between training and test sets (respectively 2/3 and 1/3 of the original dataset). The expression profile of genes selected in Step 1 (in the samples selected in the training set) is used to train a classification model to predict Cancer features. The classification accuracy of the trained model is then estimated on the test set. The classification accuracy estimated in step 2 is then compared to the classification accuracy estimated in step 1 to establish the tissue specificity of the gene signatures (**Figure 3**). In order to demonstrate the tissue specificity of models based on the molecular profile of tumour tissues we have also performed the reverse test.

The assessment of the tissue specificity of the molecular signatures obtained with the BVS procedure has been performed using a cross-validation procedure for the error estimation as described in [56].

### Analyzing the specific contribution of genes in the predictive models

Our approach, which is based on multivariate predictive models selects combination of genes to perform predict tumour features. Therefore, differential expression between sample classes may not be always indicative of the relative contribution of a gene to sample separation. Therefore, in order to graphically represent sample classification and to estimate the contribution of each gene for class distinction, we used principal component analysis (PCA). PCA reduce the original variable space in a handful of principal components (PC). A PC is defined as a weighted sum of variables (genes). The weight or loading given to a variable is interpreted as its importance. For discussion, we focused in genes having absolute loadings values larger than 0.3 (**Figure 2** **, S1, S2 and S3**). In all cases, the chosen PC (first two) show evident class separation providing further support for the association of the selected genes and the sample classes.

### Interaction networks and functional analysis of multivariate signatures: The Ingenuity Pathway Analysis (IPA) software

The gene sets represented in the populations of models selected using the GA-MLHD procedure have been analyzed using the Ingenuity Pathway Analysis (IPA) application (Palo Alto, http://www.ingenuity.com), a web based application that enables discovery, visualization, and exploration of biologically interaction networks.

Gene lists represented in the model populations developed with normal or tumor expression data to predict capsular penetration or Gleason score were uploaded into in the application. Each gene identifier was mapped to its corresponding gene object in the Ingenuity Pathways Knowledge Base. These genes, called focus genes, were overlaid onto a global molecular network developed from information contained in the Ingenuity Pathways Knowledge Base. Networks of these focus genes were then algorithmically generated based on their connectivity according to the following procedure implemented in the IPA software application. The specificity of connection for each focus gene was calculated by the percentage of its connection to other focus genes. The initiation and the growth of pathways proceed from the gene with the highest specificity of connections. Each network had a maximum of 35 genes for easier interpretation and visual inspection. Pathways of highly interconnected genes were identified by statistical likelihood using the following equation:
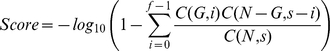



Where *N* is the number of genes in the genomic network, of which *G* are focus genes, for a pathway of *s* genes, *f* of which are focus genes. *C(n,k)* is the binomial coefficient. Pathways whose *Score* were greater than 5 (p<0.0001) were selected for biological interpretation.

Canonical pathway analysis was performed using the IPA tools and significance for the enrichment of the genes with a particular Canonical Pathway was determined by right-tailed Fisher's exact test with α = 0.01 and a whole database as a reference set.

### Analysis of LCM cell populations

The dataset developed by Tomlins et al. [11] was downloaded from the GEO database and raw data normalized using print tip normalization. The expression profiles of a subset of 20 genes (representative of secreted factors and their receptors from the IPA networks) across samples representing normal and tumour epithelial cells were then selected to create a secondary dataset. Differentially expressed genes were then identified by one factor ANOVA using the software application TMEV [57].

## Supporting Information

Figure S1 **Multivariate Models for Capsular Penetration using Tumour data.** Genes present in GA-MLHD and BVS for the same dataset are highlighted in red. Accuracy is estimated as described in the Material and Methods section. GeneBank accession number and gene symbol is shown. Brighter green or red colours in heatmaps represent lower or higher relative expression respectively. t-test p-value is shown for comparison with the differential expression criteria commonly used in univariate variable selection approaches. PCA plots and loadings are used to show the putative contribution of every gene to class separation. For example, TALDO1 gene in top heatmap seems to contribute strongly to positive Capsular Penetration whereas ST14 contribute weakly to negative Capsular Penetration. PCs were selected by visual inspection.
(EPS)

Figure S2 **Multivariate Models for Gleason Score using Normal data.** Genes present in GA-MLHD and BVS for the same dataset are highlighted in red. Accuracy is estimated as described in the Material and Methods section. GeneBank accession number and gene symbol is shown. Brighter green or red colours in heatmaps represent lower or higher relative expression respectively. t-test p-value is shown for comparison with the differential expression criteria commonly used in univariate variable selection approaches. PCA plots and loadings are used to show the putative contribution of every gene to class separation. For example, TEGT gene in top heatmap seems to contribute strongly to high Gleason grades whereas D89667 contribute to low Gleason Grades. PCs were selected by visual inspection.
(EPS)

Figure S3 **Multivariate Models for Gleason Score using Tumour data.** Genes present in GA-MLHD and BVS for the same dataset are highlighted in red. Accuracy is estimated as described in the Material and Methods section. GeneBank accession number and gene symbol is shown. Brighter green or red colours in heatmaps represent lower or higher relative expression respectively. t-test p-value is shown for comparison with the differential expression criteria commonly used in univariate variable selection approaches. PCA plots and loadings are used to show the putative contribution of every gene to class separation. For example, ACPP gene in top heatmap seems to contribute strongly to low Gleason grades whereas TM8B4X contribute to low Gleason Grades. PCs were selected by visual inspection.
(EPS)

Figure S4 **Univariate gene selection models.** Models were generated using a forward selection procedure that includes, progressively, genes ranked by a univariate statistic (F-ratio, horizontal axis). The accuracy is assessed by leave-one-out-cross-validation for a number of classification methods (vertical axis, see legends, and the Prophet tool within www.gepas.org [3]). Maximum accuracy is marked by a dotted horizontal line. Overall, this univariate gene selection generates comparable predictive models irrespective of the classification method. More accurate multivariate models generated by GA-MLHD and BVS used in this chapter are shown for comparison in red and black dots. Legends: DLDA - Diagonal Linear Discriminant Analysis, KNN - K-Nearest-Neighbours, PAMR - Shrunken Centroids, SOM - Self Organized Maps, and SVM - Support Vector Machines. See GEPAS [3] for details in F-ratio, error estimation, and classification methods. Dataset, normal or tumour data, and class is specified in each plot.
(EPS)

Figure S5 **Functional networks representing known interaction between genes expressed in normal tissue and selected in the models predictive of capsular penetration.** The figure represents the four most significant networks selected by the IPA software for the Singh et al. dataset [4]. Genes represented in the predictive models are represented by blue shapes. Genes in the networks are arranged by cellular localization (extracellular, membrane, cytoplasm and nucleus).
(TIFF)

Figure S6 **Induction of pro-metastatic cytokines in RWPE1 cells by Interleukin 1β.** The transcriptional response of normal prostate epithelial cells (RWPE1) was measured with human Agilent microarrays 6 hours and 24 hours after addition of 100 ng/ml of recombinant human Interleukin 1β (eBioscience, USA). The experiments were performed three times in different days. Genes represented in Figure 4A were then tested for differential expression using a t-test. Only the pro-metastatic chemokines CCL20 (Panel B) and CX3CL1 (Panel C) were differentially expressed (**, FDR<1%) at both time points. The gene LOX was only transiently activated by Interleukin 1β six hours post exposure (Panel D). Panel A shows the portion of the network in Figure 4A where genes are differentially expressed in RWPE1 in response to Interleukin 1β exposure. In this experiment RWPE1 cells were grown in 0.4% gelatin coated plates, complete KSFM media supplemented with L-Glutamine, p/s, BPE and EGF.
(TIFF)

Figure S7 **Expression of selected secreted factors and receptors in Tomlins et al. dataset.** Nor, Adj, BPH, PIN, PCA-Low, PCA-High and Meta samples are described in main paper.
(TIFF)

Figure S8 **Comparison of the expression of selected secreted factors and receptors in Tomlins et al. dataset.** Panels A-L represents the expression profile (y axis) of a selection of the genes differentially expressed between all LCM cell populations (shown as a heat map in figure 5 in main paper). The different cell populations are arranged along the x axis. Red close circles represent gene expression levels significantly different (P<0.01) respect to adj cells whereas blue close circles inside red circles represent gene expression levels significantly different (p<0.05) respect to adj cells. Nor, Adj, BPH, PIN, PCA-Low, PCA-High and Meta samples are described in main paper.
(TIFF)

Table S1 **Datasets annotation.** As stated, we used approximately the 25% of the database (marked in bold). Overlaps were estimated by Unigene annotation. Similar results are obtained using entrez id or gene symbol as shown in columns. 50% Top genes were estimated relaxing the filter range in both datasets to 25 and 50%.
(DOC)

Table S2 **Significant Networks identified by the Ingenuity Pathway Analysis (IPA) software associated to models developed using the GA-MLHD procedure.** The table lists the networks identified from the Lapointe et al. [2] dataset associated to models predictive of tumour capsular penetration from the molecular profile of normal cells. HCG Column highlights the network highest connected gene(s) or complex. Genes in bold were part of the multivariate models used as input for IPA analysis.
(DOC)

Table S3 **Overlap of the top 50 selected genes in models using larger datasets for Singh** ***et al.*** **dataset.** Numbers in upper triangular matrix correspond to the number of genes overlapped. Underlined numbers in lower triangular matrix correspond to the p-value testing the corresponding overlap number using a hypergeometric test. All comparisons were significant at the 0.05 level.
(DOC)

Table S4 **Selected secreted factors and receptors.** Genes obtained in IPA networks and present in Tomlins et al. dataset were selected. P-Values were estimated using f-test comparing Nor, Adj, BPH, PIN, PCA-Low, PCA-High and Meta samples as shown in Figure 5 and Supplementary Figure S6. Some genes are represented by different probes in the microarray platform used. Only probes with p-Value <0.001 were included in Figure 5.
(DOC)

Table S5 **Overlap of the top 50 selected genes in models using larger datasets for Lapointe** ***et al.*** **dataset.** Numbers in upper triangular matrix correspond to the number of genes overlapped. Underlined numbers in lower triangular matrix correspond to the p-value testing the corresponding overlap number using a hypergeometric test. All comparisons were significant at the 0.05 level.
(DOC)
