# Supplementary figures and images for: Analysis of Normal-Tumour Tissue Interaction in Tumours: Prediction of Prostate Cancer Features from the Molecular Profile of Adjacent Normal Cells
Source: PLoS One. 2011 Mar 30;6(3):e16492. doi: 10.1371/journal.pone.0016492 (PMC3068146; doi:10.1371/journal.pone.0016492)

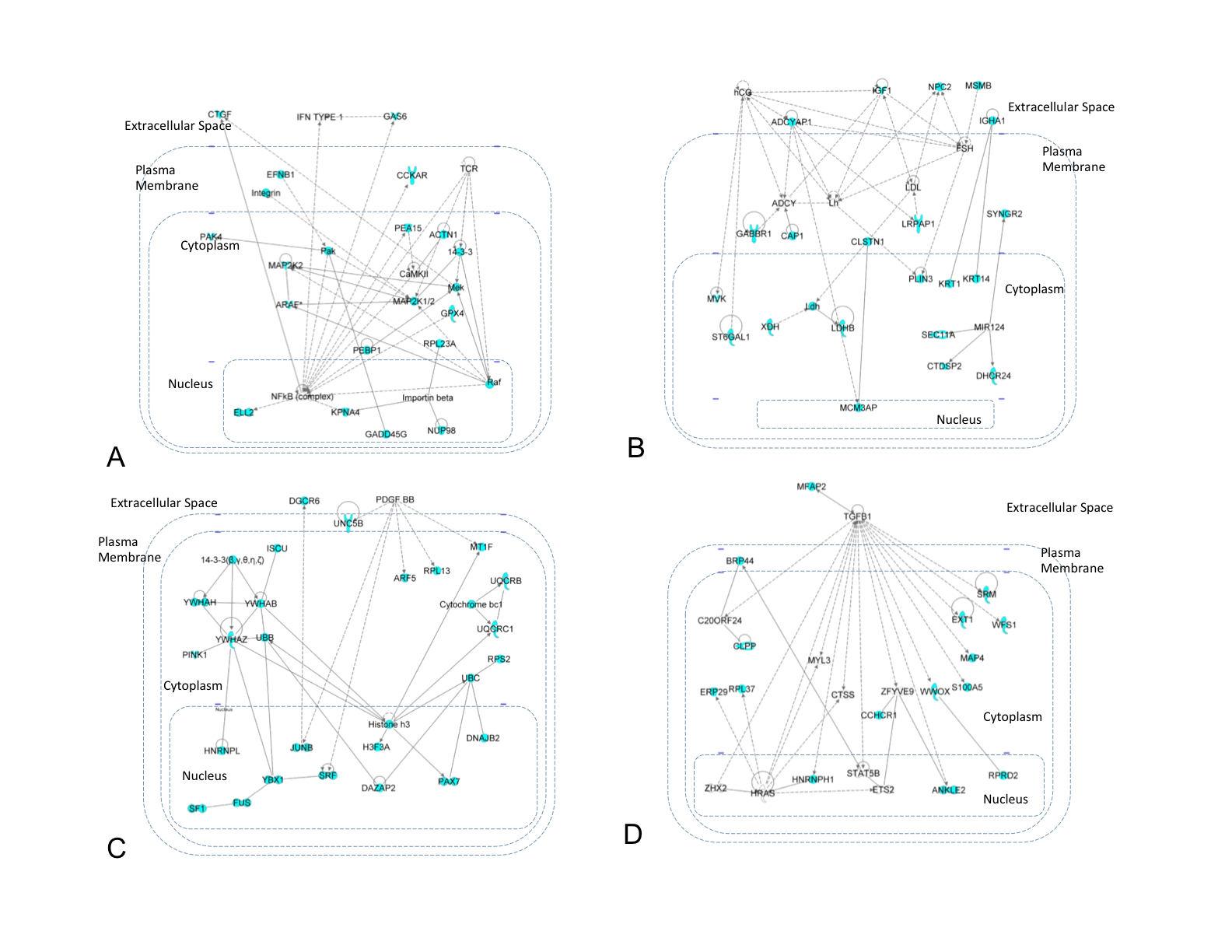

Supplement: Figure S5 — Functional networks representing known interaction between genes expressed in normal tissue and selected in the models predictive of capsular penetration. The figure represents the four most significant networks selected by the IPA software for the Singh et al. dataset [4]. Genes represented in the predictive models are represented by blue shapes. Genes in the networks are arranged by cellular localization (extracellular, membrane, cytoplasm and nucleus). (TIFF) [file pone.0016492.s005.tif]

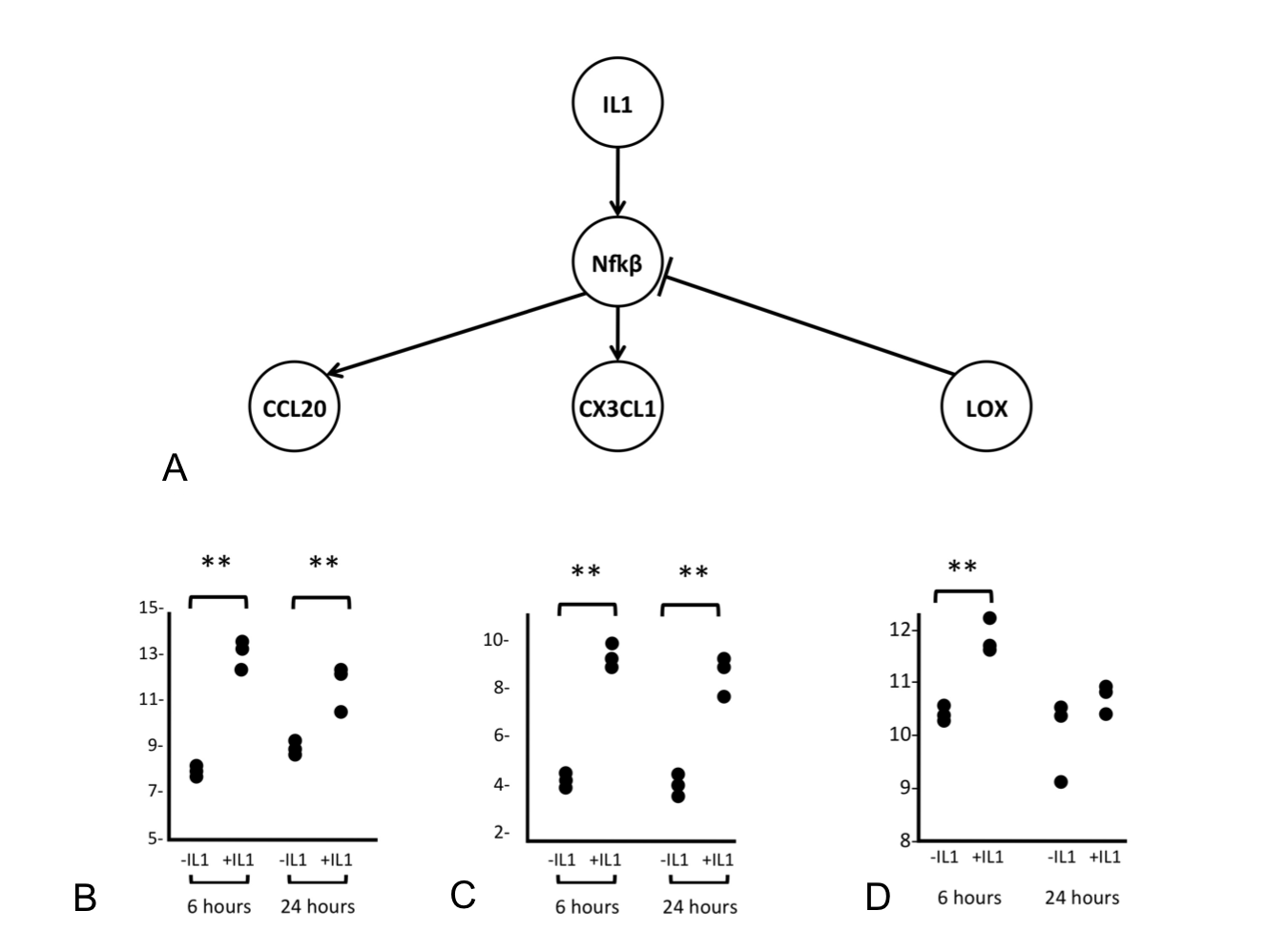

Supplement: Figure S6 — Induction of pro-metastatic cytokines in RWPE1 cells by Interleukin 1β. The transcriptional response of normal prostate epithelial cells (RWPE1) was measured with human Agilent microarrays 6 hours and 24 hours after addition of 100 ng/ml of recombinant human Interleukin 1β (eBioscience, USA). The experiments were performed three times in different days. Genes represented in Figure 4A were then tested for differential expression using a t-test. Only the pro-metastatic chemokines CCL20 (Panel B) and CX3CL1 (Panel C) were differentially expressed (**, FDR<1%) at both time points. The gene LOX was only transiently activated by Interleukin 1β six hours post exposure (Panel D). Panel A shows the portion of the network in Figure 4A where genes are differentially expressed in RWPE1 in response to Interleukin 1β exposure. In this experiment RWPE1 cells were grown in 0.4% gelatin coated plates, complete KSFM media supplemented with L-Glutamine, p/s, BPE and EGF. (TIFF) [file pone.0016492.s006.tif]

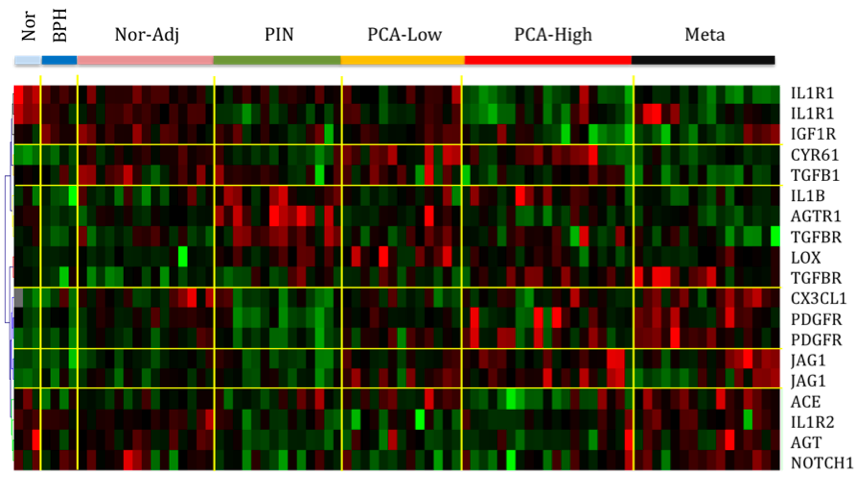

Supplement: Figure S7 — Expression of selected secreted factors and receptors in Tomlins et al. dataset. Nor, Adj, BPH, PIN, PCA-Low, PCA-High and Meta samples are described in main paper. (TIFF) [file pone.0016492.s007.tif]

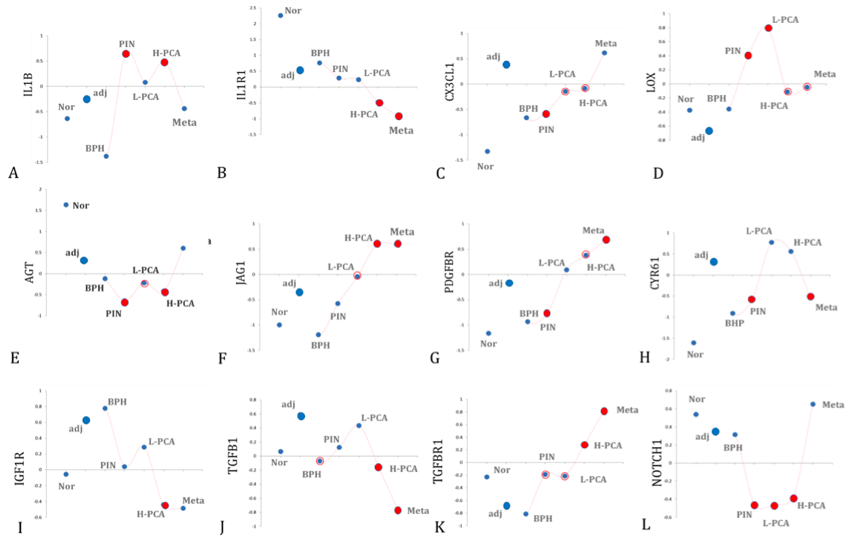

Supplement: Figure S8 — Comparison of the expression of selected secreted factors and receptors in Tomlins et al. dataset. Panels A-L represents the expression profile (y axis) of a selection of the genes differentially expressed between all LCM cell populations (shown as a heat map in figure 5 in main paper). The different cell populations are arranged along the x axis. Red close circles represent gene expression levels significantly different (P<0.01) respect to adj cells whereas blue close circles inside red circles represent gene expression levels significantly different (p<0.05) respect to adj cells. Nor, Adj, BPH, PIN, PCA-Low, PCA-High and Meta samples are described in main paper. (TIFF) [file pone.0016492.s008.tif]
