# Supplementary material for: Analysis of Normal-Tumour Tissue Interaction in Tumours: Prediction of Prostate Cancer Features from the Molecular Profile of Adjacent Normal Cells
Source: PLoS One. 2011 Mar 30;6(3):e16492. doi: 10.1371/journal.pone.0016492 (PMC3068146; doi:10.1371/journal.pone.0016492)
Supplement: Table S1 — Datasets annotation. As stated, we used approximately the 25% of the database (marked in bold). Overlaps were estimated by Unigene annotation. Similar results are obtained using entrez id or gene symbol as shown in columns. 50% Top genes were estimated relaxing the filter range in both datasets to 25 and 50%. (DOC) [file pone.0016492.s009.doc]

|  | ***Unigene*** | | ***Entrez*** | | ***Symbol*** | |
| --- | --- | --- | --- | --- | --- | --- |
|  | ***Lapointe*** | ***Singh*** | ***Lapointe*** | ***Singh*** | ***Lapointe*** | ***Singh*** |
| 100% Top Genes |  |  |  |  |  |  |
| Rows | 11490 | 12600 | 11490 | 12600 | 11490 | 12600 |
| Annotated | 9940 | 9878 | 9940 | 9589 | 7585 | 9587 |
| Overlapped | 4020 | 3961 | 4030 | 3952 | 4031 | 3950 |
| (%) | 35.0% | 31.4% | 35.1% | 31.4% | 35.1% | 31.3% |
| Unique Overlapped | 3012 | 3012 | 2995 | 2995 | 2994 | 2994 |
| (%) | 26.2% | 23.9% | 26.1% | 23.8% | 26.1% | 23.8% |
|  |  |  |  |  |  |  |
| 50% Top Genes |  |  |  |  |  |  |
| Rows | 5754 | 5190 | 5754 | 5190 | 5754 | 5190 |
| Annotated | 4887 | 3976 | 4887 | 3890 | 3515 | 3890 |
| Overlapped | 872 | 799 | 884 | 805 | 884 | 805 |
| (%) | 15.2% | 15.4% | 15.4% | 15.5% | 15.4% | 15.5% |
| Unique Overlapped | 687 | 687 | 693 | 693 | 693 | 693 |
| (%) | 11.9% | 13.2% | 12.0% | 13.4% | 12.0% | 13.4% |
|  |  |  |  |  |  |  |
| **(~25%) Top Genes (used)** | ~25% | ~25% | ~25% | ~25% | ~25% | ~25% |
| **Rows** | 2647 | 2758 | 2647 | 2758 | 2647 | 2758 |
| **Annotated** | 2261 | 2080 | 2261 | 2026 | 1617 | 2026 |
| **Overlapped** | 212 | 199 | 216 | 202 | 216 | 202 |
| **(%)** | 8.0% | 7.2% | 8.2% | 7.3% | 8.2% | 7.3% |
| **Unique Overlapped** | 174 | 174 | 177 | 177 | 177 | 177 |
| **(%)** | 6.6% | 6.3% | 6.7% | 6.4% | 6.7% | 6.4% |

**Table S1**. **Datasets annotation**. As stated, we used approximately the 25% of the database (marked in bold). Overlaps were estimated by Unigene annotation. Similar results are obtained using entrez id or gene symbol as shown in columns. 50% Top genes were estimated relaxing the filter range in both datasets to 25 and 50%.
