# Supplementary material for: Analysis of Normal-Tumour Tissue Interaction in Tumours: Prediction of Prostate Cancer Features from the Molecular Profile of Adjacent Normal Cells
Source: PLoS One. 2011 Mar 30;6(3):e16492. doi: 10.1371/journal.pone.0016492 (PMC3068146; doi:10.1371/journal.pone.0016492)
Supplement: Table S2 — Significant Networks identified by the Ingenuity Pathway Analysis (IPA) software associated to models developed using the GA-MLHD procedure. The table lists the networks identified from the Lapointe et al. [2] dataset associated to models predictive of tumour capsular penetration from the molecular profile of normal cells. HCG Column highlights the network highest connected gene(s) or complex. Genes in bold were part of the multivariate models used as input for IPA analysis. (DOC) [file pone.0016492.s010.doc]

| ***#*** | ***HCG*** | ***Molecules in Network*** | ***Score*** | ***Focus*** | ***Top Functions*** |
| --- | --- | --- | --- | --- | --- |
| 1 | NFkB | 26s Proteasome, **BEX2**, Calcineurin protein(s), Caspase, **CBLB**, **CCL20**, **CX3CL1**, **GAL**, **GHR**, Growth hormone, **GSTM2**, Hsp70, Hsp90, IFN Beta, IKK (complex), Ikk (family), IL1, **ISL1**, Jnk, **LOX**, **LPL**, **MALT1**, **MAVS**, NFkB (complex), NFkB (family), **NLRP2**, **PKP1**, **PTPN9**, **SLC2A2**, **ST8SIA1**, **TAB2**, **TP53BP2**, Trypsin, Ubiquitin, **VIPR1** | 38 | 20 | Nervous System Development and Function, Neurological Disease, Organismal Injury and Abnormalities |
| 2 | MAPK, TP53 | **AGT**, Alp, Ap1, **CCDC99**, **CCNB2**, Cdc2, **COL4A1**, Creb, Cyclin A, **CYP19A1**, **DACH1**, **DBC1**, **DCLRE1A**, E2f, Estrogen Receptor, **FANCC**, hCG, Hdac, Histone h3, Histone h4, IL12 (complex), **JAG1**, Mmp, **MUC5AC**, **MYLPF**, **NUSAP1**, **OAT**, P38 MAPK, **PLA1A**, **RUNX2**, **SH3BGRL2**, Tgf beta, **TP53**, **UBE2C**, **UTRN** | 35 | 20 | Cell Cycle, Cancer, Reproductive System Disease |
| 3 | HNF4A | CCDC59, CDK2, **CEACAM1**, CHRNA7, **CPSF7**, **CSGALNACT1**, **CTDSPL2**, Fibrinogen, FN1, **FRAS1**, **GINS2**, GRIP1, HNF4A, **ITGA8** (includes EG:8516), JUB, **MYH8**, **NAT2**, **NKX2-1**, NPNT, **OGN**, **PAG1**, **PCNP**, **PEX5L**, POMC, PRNP, **RCBTB1**, **RIC3** (includes EG:79608), RPL10, SERPINA3, SHC1, SRC, SSBP1, SYN1, TSHR, WBP4 | 28 | 16 | Developmental Disorder, Amino Acid Metabolism, Small Molecule Biochemistry |
| 4 | IGF1, MAPK, ERK, PI3K | **CCL19**, **CDO1**, **CEACAM1**, **CEACAM5** (includes EG:1048), **CNN1**, Collagen type IV, **CYR61**, ERK1/2, Focal adhesion kinase, G protein alphai, G-protein beta, **GFRA2**, **GNAI1**, **IGF1**, **IGF1R**, **ILDR1**, Laminin, LDL, Mapk, **MST1R**, p70 S6k, p85 (pik3r), Pdgf, PDGF BB, Pi3-kinase, PI3K, **PIK3CG**, PLA2, PLC gamma, Rac, Ras, **RGS5**, Shc, **VCAN**, Vegf | 25 | 15 | Cellular Growth and Proliferation, Developmental Disorder, Cellular Movement |
| 5 | MYC, CTNNB1 | ANK2, **ATP11A**, CCNG1, **CDH17**, CNP, CSDA, CTNNB1, **CYR61**, **EDIL3**, EIF4A1, **EPHB3**, **GALNT10**, MIR122 (includes EG:406906), MIR205 (includes EG:406988), MYC, **MYCT1**, NCAM1, NFYB, **OAT**, PFK, **PFKP**, Phosphofructokinase, **PPP1R3F**, PPP2CA, Pyruvate kinase, RPL19, RPL22, RPL41, RPLP2, **SGOL2**, **SSR4**, **STX16**, **SYNPO2**, **TSPAN8**, YWHAZ | 25 | 15 | Cell Death, Cardiovascular System Development and Function, Organismal Development |
| 6 | AKT, ERK | Actin, ADCY, Akt, **BCL11A**, **CDCA7**, Collagen type I, **EPHA7**, **ERAP2**, ERK, F Actin, **FSCN1**, FSH, **GRIA4**, **GRM3**, **IFI27**, IgG, **IL1RN**, Immunoglobulin, Insulin, Interferon alpha, Lh, **LRRC32**, MIR124, Mlc, **MYCN**, **NAA15**, Nfat (family), Pka, Pkc(s), PP2A, Ras homolog, **RFC3**, **RND2**, **TRIM29**, **WAC** | 23 | 16 | Cellular Assembly and Organization, Cellular Function and Maintenance, Behavior |
| 7 | PPARG | ADM, AKT2, beta-estradiol, **BEX1**, BGLAP, BTG2, **CNTN4**, dehydroepiandrosterone sulfate, EFNB2, **FABP1**, FABP4, FOXC2, FUCA1, **GATA2**, GJA1, IGFBP1, IGFBP6, **LEFTY2**, LYL1, MIR200C (includes EG:406985), MPO, NCAM1, PHLDA1, **POPDC2**, POU1F1, PPARG, RARB, RCAN1, **SEL1L3**, SLC20A1, **TMPRSS2**, **VAT1L**, VCL, **WFDC2**, **ZBTB20** | 17 | 11 | Carbohydrate Metabolism, Molecular Transport, Small Molecule Biochemistry |
| 8 | IL6, IFNG | ANK2, Caspase 3/7, **CPNE4**, CX3CR1, CXCL6, Cyclin E, Cyclooxygenase, CYP27B1, E2F1, ETV6, **GCA**, **GPR155**, **HIST1H4H** (includes EG:8365), HMGB1L1, IFNG, IFNGR2, IGFBP1, IL6, IL4R, **ITPR3**, MAP2K1, **MRVI1**, PDGF-AA, PLA2G2A, **PMP2**, PSME1, RNA polymerase II, **RNF180**, S100A10, **SIGMAR1**, SST, TK1, TNFRSF12A, UBC, ZFP36 | 13 | 9 | Cell-To-Cell Signaling and Interaction, Antigen Presentation, Hematological System Development and Function |
| 9 | HOXA10 | BCL6, beta-estradiol, BMI1, DHDDS, EIF3A, ELMOD1, **GULP1**, H2AFZ, HDLBP, HLF (includes EG:3131), **HOXA10**, HTT, ITGB3, **MEIS2**, MIR135A1, MIR135A2, MIR135B (includes EG:442891), MIR136 (includes EG:406927), MIR181B1, MIR181B2, MIR202 (includes EG:387198), MIR93 (includes EG:407050), MIRLET7B (includes EG:406884), **MS4A7**, MTPN, PBX1, PDCD10, RHOG, **SLC6A15**, **SLMAP**, SON, TSC22D3, YPEL5, ZEB2, **ZNF385B** | 10 | 7 | Cell Death, Neurological Disease, Organismal Injury and Abnormalities |

**Table S2.** **Significant Networks identified by the Ingenuity Pathway Analysis (IPA) software associated to models developed using the GA-MLHD procedure.** The table lists the networks identified from the Lapointe et al. [2] dataset associated to models predictive of tumour capsular penetration from the molecular profile of normal cells. HCG Column highlights the network highest connected gene(s) or complex. Genes in bold were part of the multivariate models used as input for IPA analysis.
