# Supplementary material for: Analysis of Normal-Tumour Tissue Interaction in Tumours: Prediction of Prostate Cancer Features from the Molecular Profile of Adjacent Normal Cells
Source: PLoS One. 2011 Mar 30;6(3):e16492. doi: 10.1371/journal.pone.0016492 (PMC3068146; doi:10.1371/journal.pone.0016492)
Supplement: Table S3 — Overlap of the top 50 selected genes in models using larger datasets for Singh et al. dataset. Numbers in upper triangular matrix correspond to the number of genes overlapped. Underlined numbers in lower triangular matrix correspond to the p-value testing the corresponding overlap number using a hypergeometric test. All comparisons were significant at the 0.05 level. (DOC) [file pone.0016492.s011.doc]

| Gene | p value |
| --- | --- |
| IL1R1 | 2.61E-10 |
| AGTR | 2.36E-08 |
| CYR61 | 6.37E-08 |
| JAG1 | 1.48E-05 |
| PDGFR[AB] | 3.28E-05 |
| AGT | 7.63E-05 |
| IL1R1 | 0.000147 |
| TGFBR | 0.000160 |
| JAG1 | 0.000185 |
| IL1B | 0.000269 |
| PDGFR[AB] | 0.001057 |
| NOTCH1 | 0.002586 |
| TGFBR | 0.002608 |
| CX3CL1 | 0.002813 |
| TGFB1 | 0.002876 |
| IL1R2 | 0.003042 |
| LOX | 0.003289 |
| IGF1R | 0.005362 |
| ACE | 0.006347 |
| TGFBR | 0.027028 |
| TGFBR | 0.029577 |
| IGF1 | 0.059573 |
| REN | 0.079455 |
| CXCR6 | 0.095177 |
| NOTCH2 | 0.217420 |
| CX3CR1 | 0.255131 |
| PDGFB | 0.448600 |
| PDGFB | 0.922870 |

**Table S3.** **Selected secreted factors and receptors.** Genes obtained in IPA networks and present in Tomlins et al. dataset were selected. P-Values were estimated using f-test comparing Nor, Adj, BPH, PIN, PCA-Low, PCA-High and Meta samples as shown in Figure 5 and Supplementary Figure 6. Some genes are represented by different probes in the microarray platform used. Only probes with p-Value < 0.001 were included in Figure 5.
