# Supplementary material for: Analysis of Normal-Tumour Tissue Interaction in Tumours: Prediction of Prostate Cancer Features from the Molecular Profile of Adjacent Normal Cells
Source: PLoS One. 2011 Mar 30;6(3):e16492. doi: 10.1371/journal.pone.0016492 (PMC3068146; doi:10.1371/journal.pone.0016492)
Supplement: Table S4 — Selected secreted factors and receptors. Genes obtained in IPA networks and present in Tomlins et al. dataset were selected. P-Values were estimated using f-test comparing Nor, Adj, BPH, PIN, PCA-Low, PCA-High and Meta samples as shown in Figure 5 and Supplementary Figure S6. Some genes are represented by different probes in the microarray platform used. Only probes with p-Value <0.001 were included in Figure 5. (DOC) [file pone.0016492.s012.doc]

|  | ***Top 28% (2759)*** | *Top 50% (5191)* | *Top 100% (12600)* |
| --- | --- | --- | --- |
| Gleason Score Tumor |  |  |  |
| **Top 28% (2759)** | - | 18 | 11 |
| Top 50% (5191) | 3.61E-22 | - | 12 |
| Top 100% (12600) | 2.28E-11 | 3.13E-16 | - |
|  |  |  |  |
| Gleason Score Normal |  |  |  |
| **Top 28% (2759)** | - | 14 | 5 |
| Top 50% (5191) | 1.10E-15 | - | 10 |
| Top 100% (12600) | 2.26E-04 | 5.82E-13 | - |
|  |  |  |  |
| Capsular Penetration Tumor |  |  |  |
| **Top 28% (2759)** | - | 13 | 7 |
| Top 50% (5191) | 3.43E-14 | - | 18 |
| Top 100% (12600) | 1.97E-06 | 2.50E-27 | - |
|  |  |  |  |
| Capsular Penetration Normal |  |  |  |
| **Top 28% (2759)** | - | 13 | 4 |
| Top 50% (5191) | 3.43E-14 | - | 7 |
| Top 100% (12600) | 1.83E-03 | 1.64E-08 | - |

**Table S4. Overlap of the top 50 selected genes in models using larger datasets for Singh et al. dataset.** Numbers in upper triangular matrix correspond to the number of genes overlapped. Underlined numbers in lower triangular matrix correspond to the p-value testing the corresponding overlap number using a hypergeometric test. All comparisons were significant at the 0.05 level.
