# Supplementary material for: Analysis of Normal-Tumour Tissue Interaction in Tumours: Prediction of Prostate Cancer Features from the Molecular Profile of Adjacent Normal Cells
Source: PLoS One. 2011 Mar 30;6(3):e16492. doi: 10.1371/journal.pone.0016492 (PMC3068146; doi:10.1371/journal.pone.0016492)
Supplement: Table S5 — Overlap of the top 50 selected genes in models using larger datasets for Lapointe et al. dataset. Numbers in upper triangular matrix correspond to the number of genes overlapped. Underlined numbers in lower triangular matrix correspond to the p-value testing the corresponding overlap number using a hypergeometric test. All comparisons were significant at the 0.05 level. (DOC) [file pone.0016492.s013.doc]

|  | ***Top 28% (2759)*** | *Top 50% (5191)* | *Top 100% (12600)* |
| --- | --- | --- | --- |
| Gleason Score Tumor |  |  |  |
| **Top 23% (2647)** | - | 11 | 4 |
| Top 50% (5754) | 3.67E-11 | - | 19 |
| Top 100% (11490) | 2.19E-03 | 3.04E-30 | - |
|  |  |  |  |
| Gleason Score Normal |  |  |  |
| **Top 23% (2647)** | - | 21 | 10 |
| Top 50% (5754) | 3.62E-27 | - | 20 |
| Top 100% (11490) | 7.45E-10 | 2.29E-32 | - |
|  |  |  |  |
| Capsular Penetration Tumor |  |  |  |
| **Top 23% (2647)** | - | 5 | 7 |
| Top 50% (5754) | 2.82E-04 | - | 7 |
| Top 100% (11490) | 2.67E-06 | 7.39E-09 | - |
|  |  |  |  |
| Capsular Penetration Normal |  |  |  |
| **Top 23% (2647)** | - | 14 | 10 |
| Top 50% (5754) | 2.02E-15 | - | 14 |
| Top 100% (11490) | 7.45E-10 | 2.20E-20 | - |

**Table S5. Overlap of the top 50 selected genes in models using larger datasets for Lapointe et al. dataset.** Numbers in upper triangular matrix correspond to the number of genes overlapped. Underlined numbers in lower triangular matrix correspond to the p-value testing the corresponding overlap number using a hypergeometric test. All comparisons were significant at the 0.05 level.
